# Supplementary material for: Inflammation and immune activation are associated with risk of Mycobacterium tuberculosis infection in BCG-vaccinated infants
Source: Nat Commun. 2022 Nov 3;13:6594. doi: 10.1038/s41467-022-34061-7 (PMC9632577; doi:10.1038/s41467-022-34061-7)
Supplement: Supplementary file 1 — Supplementary Information [file 41467_2022_34061_MOESM1_ESM.pdf]

Supplementary Information

Inflammation and immune activation are associated with risk of *Mycobacterium tuberculosis* infection in BCG-vaccinated infants

Iman Satti[1], Rachel E. Wittenberg[1]*, Shuailin Li[1]*, Stephanie A. Harris[1], Rachel Tanner[1], Deniz Cizmeci[1], Ashley Jacobs[15], Nicola Williams[2], Humphrey Mulenga[3], Helen A Fletcher[4], Thomas J. Scriba[3], Michele Tameris[3], Mark Hatherill[3] and Helen McShane[1]**

[1]Jenner Institute, Nuffield Department of Medicine, University of Oxford, Oxford, OX3 7DQ, UK.

[2] Nuffield Department of Primary Care Health Sciences, University of Oxford, Radcliffe Observatory Quarter, Woodstock Road, Oxford OX2 6GG, UK.

[3] South African Tuberculosis Vaccine Initiative (SATVI), Institute of Infectious Disease and Molecular Medicine and Division of Immunology, Department of Pathology, University of Cape Town, Cape Town, South Africa.

[4] Department of Infection Biology, Faculty of Infectious and Tropical Diseases, London School of Hygiene & Tropical Medicine, London, UK

[5] Wellcome Centre for Infectious Diseases Research in Africa, University of Cape Town, Observatory, Cape Town, South Africa.

* Equal contribution

** Corresponding author

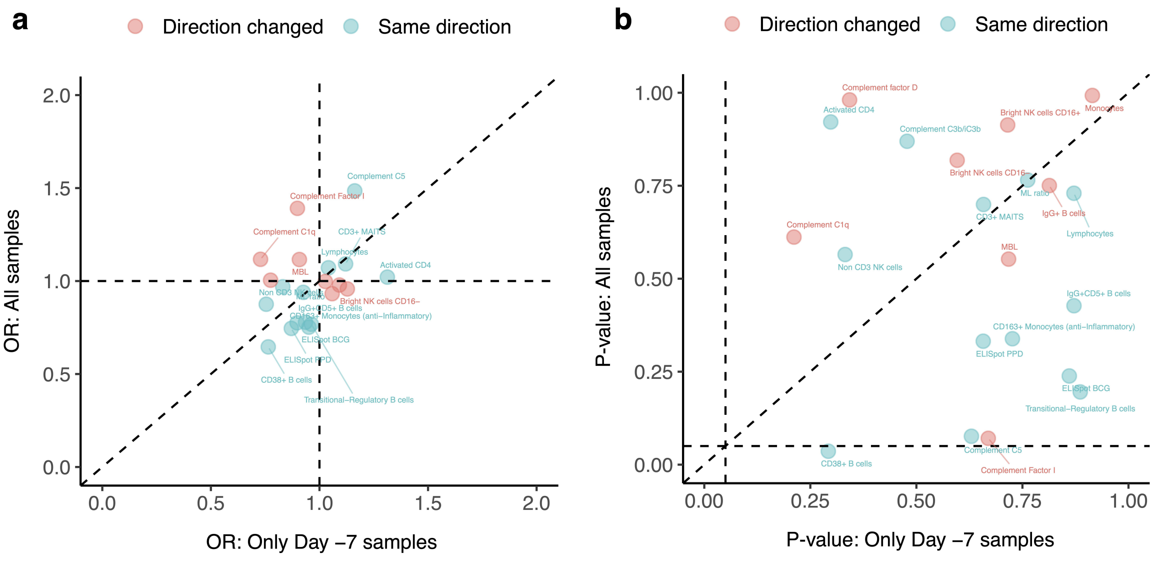

Supplementary Fig. 1: The influence of time point of sample collection on the results of conditional logistic regression.

a: Odds ratio of conditional logistic regression when Day -7 and Day 28 samples were combined and that when only Day -7 samples were included. Only immune parameters with *p*-values less than 0.1 when compared Day -7 and Day 28 samples from *M.tb*-infected infants were included. Red points represent immune parameters with qualitative different odds ratio in the two analyses (either changed from less than 1 when Day -7 and Day 28 samples combined to larger than 1 when only Day -7 samples are included or vice versa), blue points represent those with a qualitatively equal odds ratio in the two analyses.

b: Statistical significance of conditional logistic regression when Day -7 and Day 28 samples were combined and that when only Day -7 samples were included. Two-sided *P*-values were not adjusted for multiple testing correction in this figure. Colours of the points have the same meaning as those in **a**. Source data are provided in Supplementary Data 4.

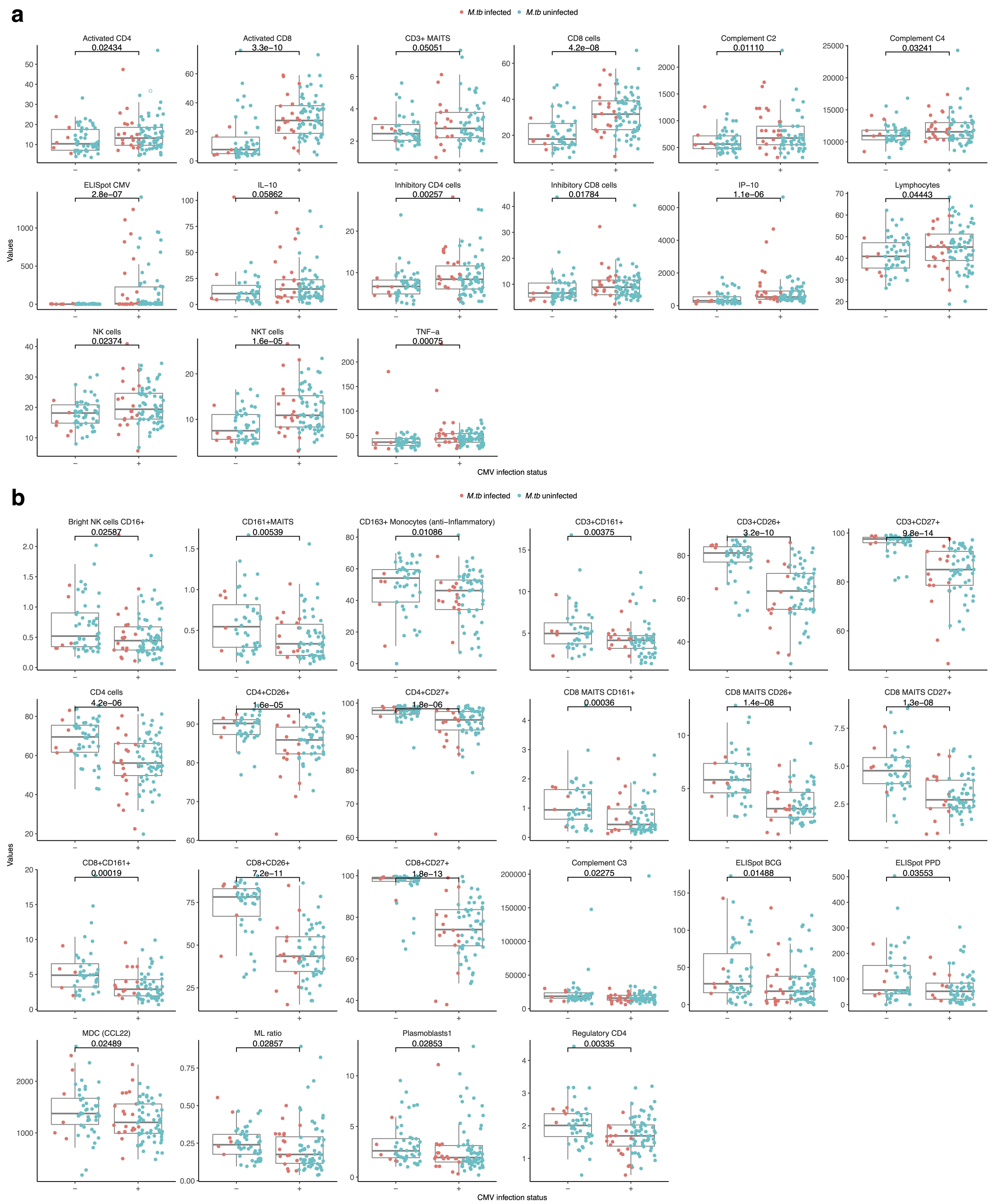

Supplementary Fig. 2: Immune parameters that differ between CMV-infected and CMV-uninfected infants.

a: Upregulated immune parameters in CMV-infected infants.

b: Downregulated immune parameters in CMV-infected infants.

The comparison of immune parameters between CMV-infected and CMV-uninfected infants was done by two-sided Mann-Whitney test. *P*-values shown were unadjusted and only immune parameters with an FDR of no more than 0.2 are shown here. Bars show medians

with the interquartile ranges (IQR). the upper whisker extends to the largest value no further than 1.5× IQR from the hinge, the lower whisker extends from the hinge to the smallest value at most 1.5× IQR from the hinge. The complete results are shown in Supplementary Data 5. The number of biologically independent samples are included in Supplementary Data 5. Source data are provided as a Source Data file.

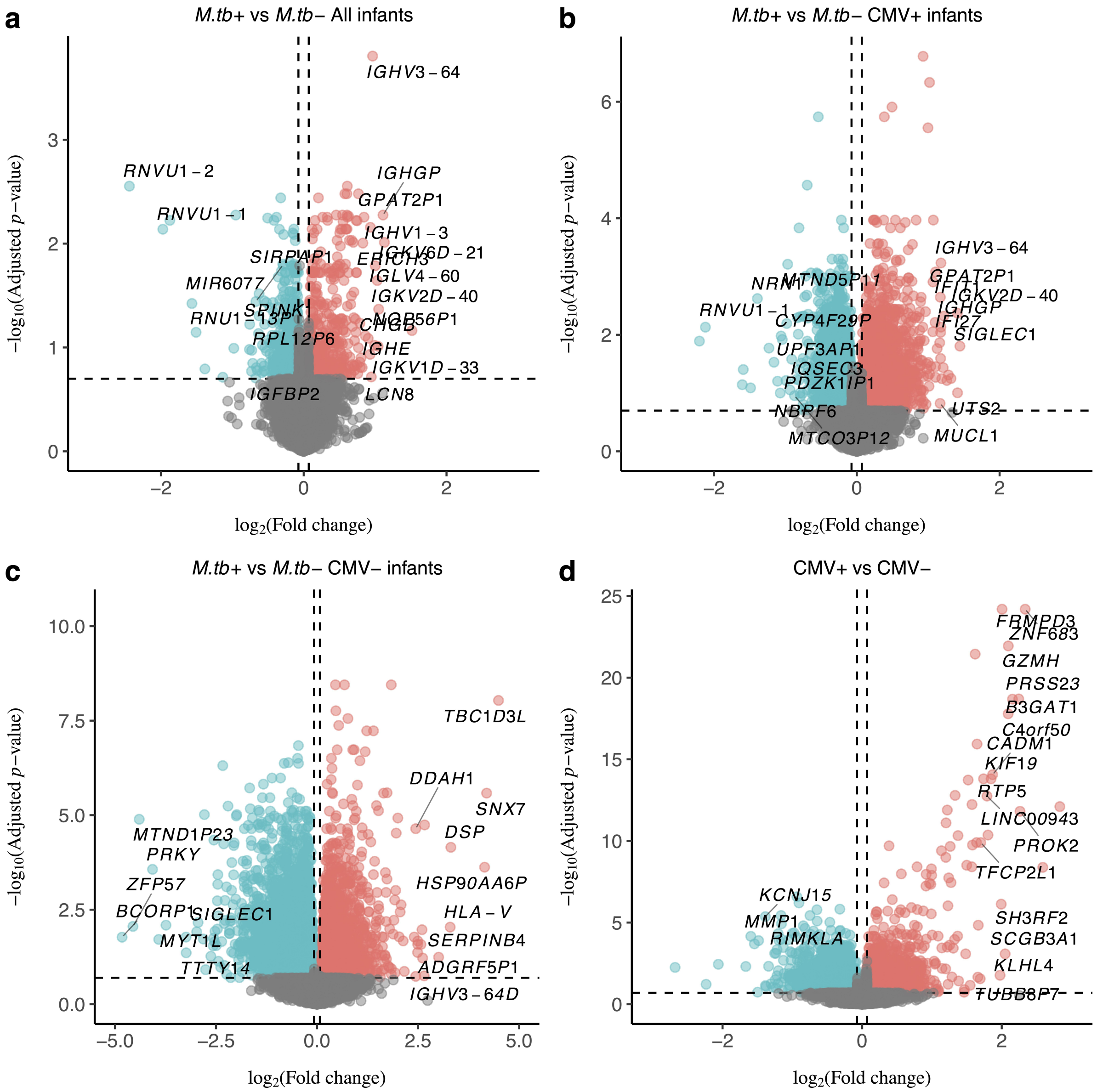

Supplementary Fig. 3: Differentially expressed genes (DEGs) between BCG-vaccinated infants who got infected with *M.tb* during the study follow-up period (*M.tb*+) and those who remained non-infected (*M.tb*-) (a-c) or between CMV-infected (CMV+) and CMV-uninfected (CMV-) infants at the beginning of the study (d).

DEGs have adjusted *p*-value < 0.2 (adjusted by Benjamini-Hochberg correction) and fold change > 1.05 or < 0.95. The top significant genes are labelled in each figure, and horizontal and vertical dashed lines indicate 20% FDR and 5% change in gene expression, respectively.

a: DEGs between infants who were subsequently *M.tb*-infected and *M.tb*-uninfected in all study subjects

b: DEGs between infants who were subsequently *M.tb*-infected and *M.tb*-uninfected in CMV-infected infants

c: DEGs between infants who were subsequently *M.tb*-infected and *M.tb*-uninfected in CMV-uninfected infants

d: DEGs between CMV-infected and CMV-uninfected infants

Red: upregulated genes. Blue: downregulated genes. Grey: non-differentially expressed genes. Two-tailed Wald test was used to calculate *P*-values. *P*-values from the subset of genes that passed the independent filtering step were adjusted using Benjamini-Hochberg multiple testing correction. Source data are provided in Supplementary Data 6.

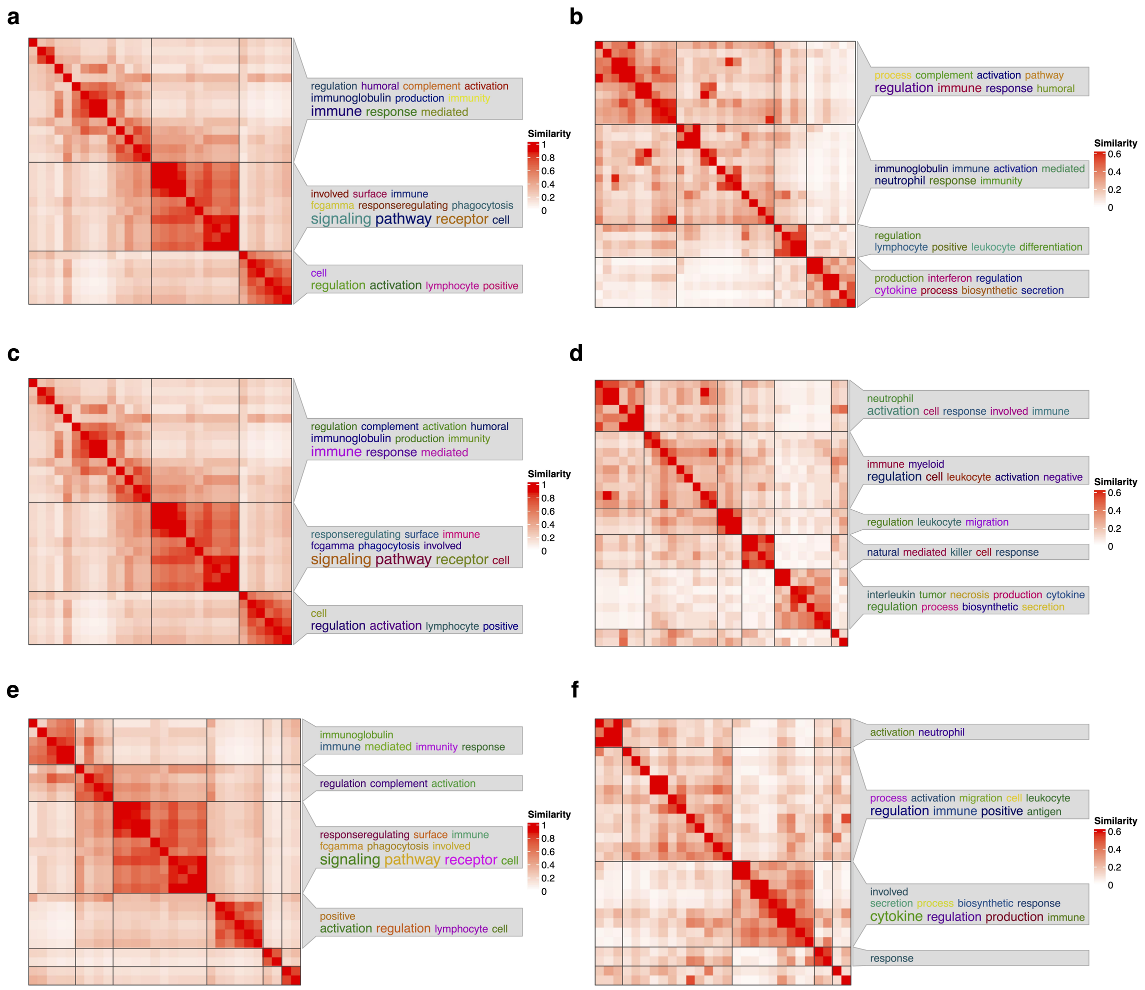

Supplementary Fig. 4: Gene sets enrichment results using overrepresentation test and gene sets defined by GO database.

Each row and column represents a gene set. The colour intensity shows semantic similarity between gene sets, which is measured by a similarity measure introduced by Wang *et. al.* 2007 [1] and calculated using GOSemSim (v2.16.1) package in R [2]. Gene sets are clustered according to their similarities. The right side of the heatmap shows the word cloud annotations, which summarize GO terms in each GO cluster with keywords. No word cloud is included for the clusters that are merged from small clusters (size < 2). The figure was drawn using simplifyEnrichment package (v.1.0.0) in R with cutoff set as 0.75 [3]. Downregulated gene sets in infants who were subsequently *M.tb*-infected, compared to *M.tb*-uninfected (All infants) and downregulated gene sets in infants who were subsequently *M.tb*-infected , compared to *M.tb*-uninfected (CMV-infected infants) are not shown because the number of enriched gene sets in these two situation is less than 2.

a. Upregulated gene sets in infants who were subsequently *M.tb* infected, compared to *M.tb*-uninfected (All infants)

b. Upregulated gene sets in infants who were subsequently *M.tb* infected, compared to *M.tb*-uninfected (CMV-infected infants)

c. Upregulated gene sets in infants who were subsequently *M.tb* infected, compared to *M.tb*-uninfected (CMV-uninfected infants)

d. Downregulated gene sets in infants who were subsequently *M.tb* infected, compared to *M.tb*-uninfected (CMV-uninfected infants)

e. Upregulated gene sets in CMV-infected infants, compared to CMV-uninfected infants

f. Downregulated gene sets in CMV-infected infants, compared to CMV-uninfected infants. Source data are provided as a Source Data file.

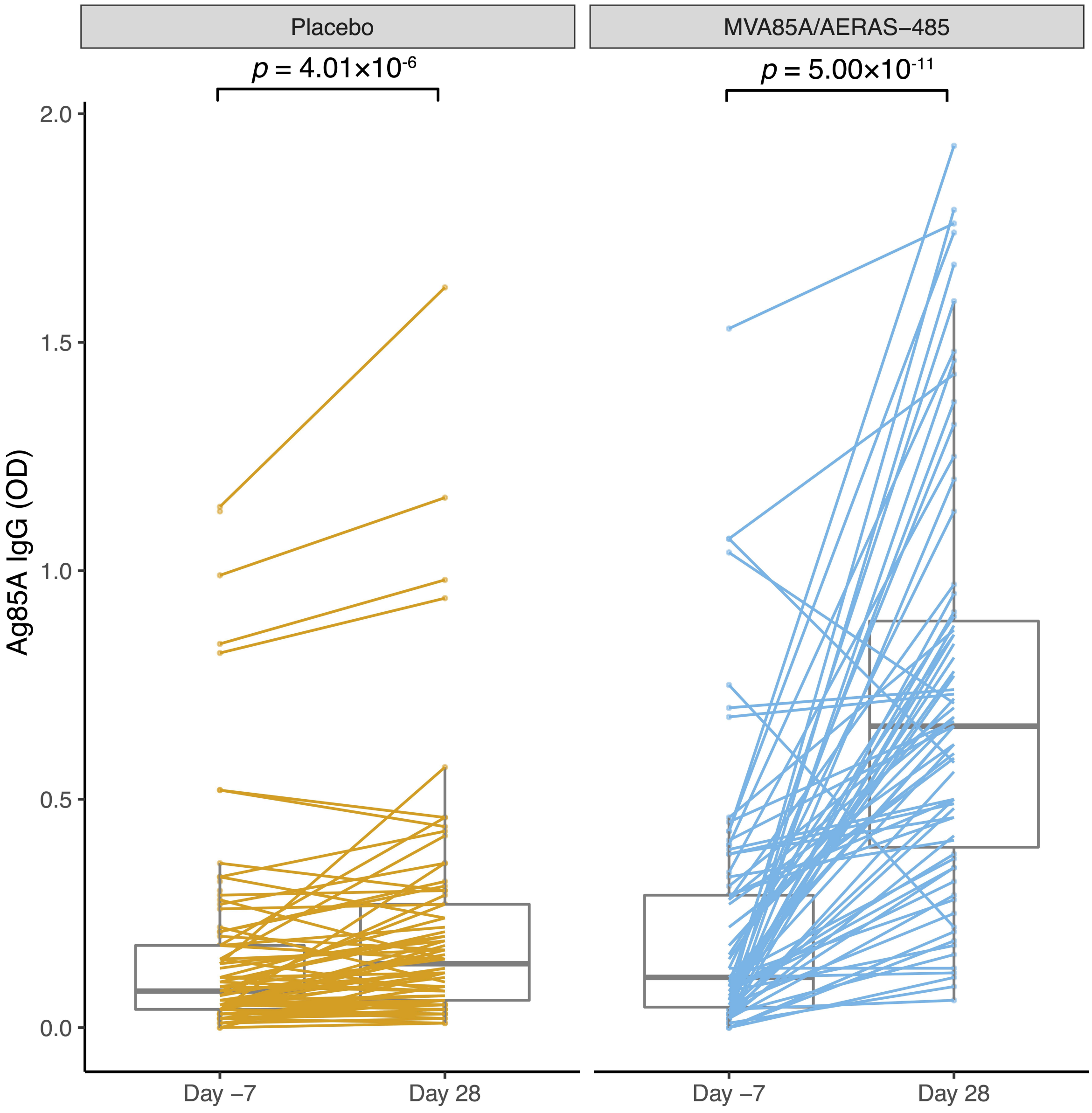

Supplementary Fig. 5: Ag85A-specific IgG response on Day -7 and Day 28 in the placebo group (left) and MVA85A-vaccinated group (right).

Bars show medians with the interquartile ranges (IQR). the upper whisker extends to the largest value no further than 1.5× IQR from the hinge, the lower whisker extends from the hinge to the smallest value at most 1.5× IQR from the hinge. N = 72 and 70 biologically independent samples for the placebo group and MVA85A-vaccinated group, respectively. Two-sided Wilcoxon test was used for comparison and *p*-values were not adjusted by multiple testing correction. Source data are provided as a Source Data file. Yellow: Placebo. Blue: MVA85A/AERAS-485.

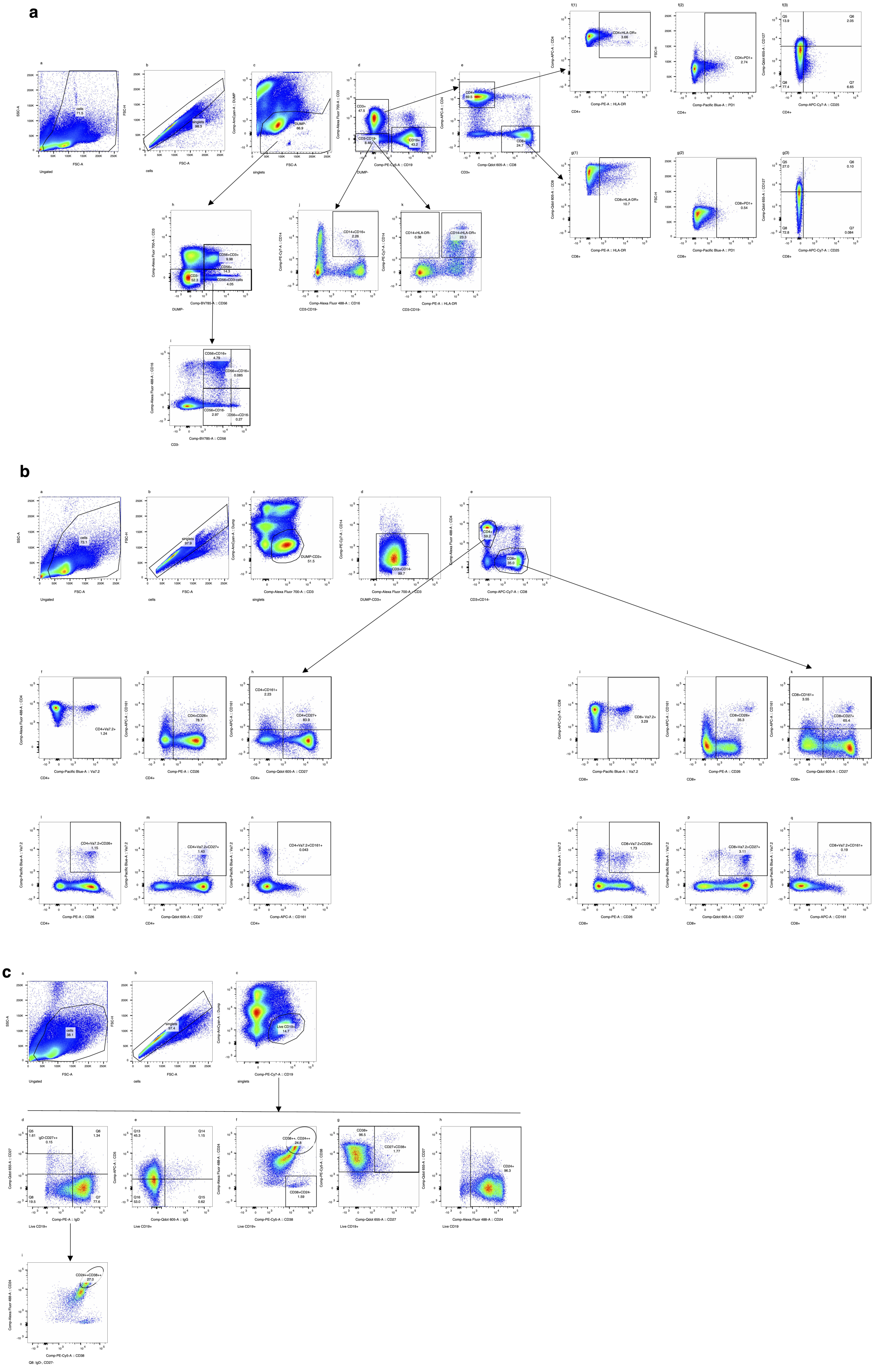

Supplementary Fig. 6: Flow cytometry gating strategies.

a: T-cells: Singlet, live cells (a-c) were included in analysis. $CD3^+$ cells (d) were used to gate $CD4^+$ and $CD8^+$ T-cells (e). The expression of HLA-DR (1), PD1 (2) and CD25/CD127 (3) was measured on $CD4^+$ (f) and $CD8^+$ (g) T-cells. CD56+, $CD56^+CD3^-$ and $CD56^+CD3^+$ were gated as shown in (h), CD16/CD56 gating is shown in (i). CD14/CD16 populations gating is shown in (j) and expression of HLA-DR on CD14 is shown in (k).
b: MAIT-cells: Singlet, live $CD14^-CD3^+$ T-cells (a-c) were included in analysis. $CD3^+$ cells (d) were used to gate $CD4^+$ and $CD8^+$ T-cells (e). Surface expression of Va7.2, CD26, CD27 and CD161 are shown in plots (f)-(h) and (i)-(k) for $CD4^+$ and $CD8^+$ T-cells respectively. Double positive Va7.2/CD26, Va7.2/CD27 and Va7.2/CD161 are shown in (l)-(n) and (o)-(q) for $CD4^+$ and $CD8^+$ T-cells respectively.
c: B-cells: Singlet, live $CD19^+$ cells (a-c) were included in analysis. B-cell populations were gated based on the expression of CD27, IgD, IgG, CD5, CD24 and CD38 as shown in plots (d)-(i), details of the subpopulation identification was based on previous studies of B-cell phenotype characterization [4–7].

Supplementary References

1. Wang, J. Z., Du, Z., Payattakool, R., Yu, P. S. & Chen, C. F. A new method to measure the semantic similarity of GO terms. *Bioinformatics* **23**, 1274–1281 (2007).
2. Yu, G. *et al.* GOSemSim: an R package for measuring semantic similarity among GO terms and gene products. *Bioinformatics* **26**, 976–978 (2010).
3. Gu, Z. & Hübschmann, D. Simplify enrichment: A bioconductor package for clustering and visualizing functional enrichment results. *Genomics Proteomics Bioinformatics* S1672022922000730 (2022) doi:10.1016/j.gpb.2022.04.008.
4. Lundell, A.-C. *et al.* High Proportion of CD5 $^+$ B Cells in Infants Predicts Development of Allergic Disease. *J. Immunol.* **193**, 510–518 (2014).
5. Maecker, H. T., McCoy, J. P. & Nussenblatt, R. Standardizing immunophenotyping for the Human Immunology Project. *Nat. Rev. Immunol.* **12**, 191–200 (2012).
6. Mauri, C. & Bosma, A. Immune Regulatory Function of B Cells. *Annu. Rev. Immunol.* **30**, 221–241 (2012).
7. Wei, C., Jung, J. & Sanz, I. OMIP-003: Phenotypic analysis of human memory B cells. *Cytometry A* **79A**, 894–896 (2011).

STATISTICAL ANALYSIS PLAN FOR IMMUNE CORRELATES OF RISK OF *MYCOBACTERIUM TUBERCULOSIS* INFECTION

Investigational Product: MVA85A/AERAS-485
Protocol Number: Oxford TB020 (Aeras C-020-485)

Version 3.0
10th April 2018

Name	Title	Signature	Date
Helen McShane	Professor of Vaccinology. Principal Investigator		
Iman Satti	Senior Scientist		
Nicola Williams	Senior Trial Statistician		

LIST OF ABBREVIATIONS

ELISA	Enzyme-linked Immunosorbent Assay
ESAT-6	6 kDA early secretory antigenic target
FDA	Food and Drug Administration
GIT	Gold In-Tube (in reference to QuantiFERON)
HIV	human immunodeficiency virus
IFN-γ	Interferon-gamma
IGRA	Interferon-gamma release assay
LTBI	Latent Tuberculosis Infection
M.tb	*Mycobacterium tuberculosis*
PBMC	Peripheral Blood Mononuclear Cells
ROC	receiver operating characteristic
SAP	Statistical Analysis Plan
TB	Tuberculosis

Table of Contents

1. INTRODUCTION AND BACKGROUND 4

2. STUDY DESIGN 5

2.1 Study Objectives 5

2.2 Scope of this analysis plan 5

2.3 Selection of assays for Correlates study 5

2.4 Sample size considerations 14

2.5 Assay methodology and blinding 14

3 STATISTICAL METHODS 14

3.1 Preliminary data review 14

3.2 Variables to be included in the model to answer the primary objective 16

3.3 Selection of variables to be included in the model to answer the secondary objective 16

3.3.2 Multivariate analysis 17

4 Computer methods....................18

5 Validation and Data Storage 18

Appendixes 19

Appendix 1: Summary of matched control selections 19

Appendix 2: Summary of C-020-485 immune correlates assays 20

IFN-γ ELISPOT assay for the detection of an antigen-specific immune responses....................20

Gene expression analysis (GEX) 21

Mycobacterial growth inhibition assay (MGIA)....................21

Flow surface staining....................22

Enzyme-linked Immunosorbent Assay (ELISA)....................23

Multiplex Assay (Luminex)....................23

Appendix 3: Data Handling....................23

References 24

1. INTRODUCTION AND BACKGROUND

An immune correlate of risk is defined as an immune marker which may be statistically correlated with the risk of development of *Mycobacterium tuberculosis* (*M.tb*) infection (predictive of infection). The immune correlate may be mechanistically responsible for infection (causal) or may not be mechanistically responsible for infection (correlative). Using the models described below we hope to identify the best performing immune marker or combination of immune markers able to predict the subsequent infection with *M.tb* in the following 1-3 years of life.

Despite universal BCG vaccination within the first 48 hours of life the annual incidence of TB disease in children under 2 years of age remains high at ~1.5% per annum in the Western Cape of South Africa [1]. New vaccines and vaccine strategies are being developed to increase protection from TB disease and *M.tb* infection through enhancement of a mycobacterial antigen specific Th1 type cellular immune response. One such strategy is to use MVA85A, a viral vector vaccine encoding the Ag85A protein from *M.tb*, to enhance an immune response primed by the BCG vaccine.

In Study C-020-485, a Phase II double-blinded randomized controlled trial for evaluation of safety, immunogenicity and efficacy of MVA85A in BCG vaccinated infants without TB or HIV, 2797 infants (age 126 to 182 days) were randomized to receive MVA85A or *Candida* skin test antigen control [2]. The study was performed in the Western Cape of South Africa. All infants were followed for at least 15 months after the last infant was enrolled into the study. The total duration of follow-up for each infant was at least 15 months and up to 39 months. Infants were followed for the entire duration both for the development of TB disease, *M.tb* infection and serious adverse events. A case of *M.tb* infection was defined by the development of a positive quantiferon test. During this efficacy trial, blood samples were collected from infants for safety, immunogenicity and immune correlates analysis.

The results of Study C-020-485 were published by Tameris *et al* in February 2013 [2]. We found no efficacy of MVA85A against TB disease (VE 17.3%; 95% CI -31.9 to 48.2) or *M.tb* infection (VE -3.8%; 95% CI - 28.1 to 15.9). As there was no efficacy with MVA85A, a planned follow-up study is not able to use stored blood samples to identify immune correlates of protection from TB disease or *M.tb* infection. However, as 349 infants were identified as *M.tb* infection cases based on the study definition, this study is therefore able to evaluate immune correlates of risk of *M.tb* infection in infants vaccinated with BCG only and/or vaccinated with BCG and boosted with MVA85A.

This case-control study will use stored blood samples from C-020-485 to produce an analysis of a set of matched controls (see appendix 1 for description of controls selection) to evaluate immune correlates of risk of *M.tb* infection for 43 cases of *M.tb* infection. This is the total number of *M.tb* infection cases available once infants with TB disease and infants receiving chemoprophylaxis have been excluded.

2. STUDY DESIGN

2.1 Study Objectives

Primary objective

The primary objective of this study is to identify correlates of risk of *M.tb* infection in BCG-vaccinated, HIV-negative infants. Specifically, the primary objective is to assess the ability of a set of three selected variables: Activated CD4+ T cells, BCG-Specific Elispot responses and Ag85A IgG, measuring immune response at Study Day -7 to predict the occurrence of *M.tb* infection (protocol definition) during study follow-up in a set of 43 *M.tb* infection cases and 3 matched controls per case.

Exploratory objective

To identify a secondary set of variables measuring immune response at Study Day -7 that best classify subjects in terms of their risk of *M.tb* infection.

2.2 Scope of this analysis plan

This statistical analysis plan (SAP) provides a summary of the analyses in support of the correlates of risk of *M.tb* infection for Study C-020-485. Specifically, this SAP contains the following:

Description of the primary, and exploratory analyses and models which will be performed to examine immune assay response, to assess the ability of these immune response measurements to predict the occurrence of *M.tb* infection during study follow-up.

Details of these analyses were not prespecified in the C-020-485 study protocol. This SAP will be finalized and signed off prior to performing the primary analyses described in Section 3. All decisions regarding model selection and refinement (e.g., inclusion of interaction terms) which are not prespecified in this SAP will be documented in a separate memorandum. All analysis will be undertaken by an independent statistician.

2.3 Selection of assays for Correlates study

Assays for this study are based on those described by Fletcher *et al*, 2016 [3], which was based on pilot studies reported in Harris *et al*, 2014 [4].
As we do not know the immune correlates of risk, the aim of these pilot experiments was to select a panel of complementary but non-overlapping immune parameters able to measure different aspects of the host immune response. In the context of these experiments, non-overlapping is defined as measuring different components of the immune response, and using

assays which do not correlate with each other. In addition to being non-overlapping, the assays had to be sensitive enough to detect an antigen specific immune response where measured, and to be reliable enough to give similar responses in replicate tests and when used repeatedly in the same individual over time (inter and intra assay reliability). The sensitivity of each assay was assessed by their ability to detect a significant increase in antigen specific immune response at day 28 following immunization with MVA85A when compared to the pre-vaccination response or to placebo (Wilcoxon $p<0.05$).

Table 1. Assay identification for C-020-485 case-control correlates analysis: Intermediate selection of key assays/variables

*these assays form the primary analysis

No.	Assay	Antigen/Cell population/cytokine	Column	Notes	Read out
1	ELISPOT	ELISpot BCG	Q	SFC/million PBMC	Primary
2	ELISPOT	ELISpot CMV	R	SFC/million PBMC	Exploratory
3	ELISPOT	ELISpot EBV	S	SFC/million PBMC	Exploratory
4	ELISPOT	ELISpot PPD	T	SFC/million PBMC	Exploratory
5	MGIA (1x10^6 cells)	MGIA 1x10^6	U	Log growth in sample tube/log growth in control tube (possible measure of vaccine response)	Exploratory
6	MGIA (3x10^6 cells)	MGIA 3x10^6	V	Log growth in sample tube/log growth in control tube (possible measure of vaccine response)	Exploratory
7	ELISA	Ag85A IgG (D0)	W	405 OD	Exploratory
8	ELISA	WCL IgG (D0)	X	405 OD	Exploratory
9	ELISA	Ag85A IgG (D28)	Y	405 OD	Primary
10	ELISA	WCL IgG (D28)	Z	405 OD	Exploratory
11	Flow Cytometry	Lymphocytes	AA	CD3,%P	Exploratory
12	Flow Cytometry	CD4 cells	AB	CD3/CD4,%P	Exploratory
13	Flow Cytometry	Activated CD4	AC	CD3/CD4/CD4+HLADR+,%P	Primary
14	Flow Cytometry	Regulatory CD4	AD	CD3/CD4/Q7: CD25+, CD127-,%P	Exploratory
15	Flow Cytometry	CD8 cells	AE	CD3/CD8,%P	Exploratory
16	Flow Cytometry	Activated CD8	AF	CD3/CD8/CD8+HLADR+,%P	Exploratory
17	Flow Cytometry	Regulatory CD8	AG	CD3/CD8/Q7: CD25+, CD127-,%P	Exploratory

18	Flow Cytometry	Non activated monocytes (suppressors)	AH	CD3-CD19-/CD14+HLADR-,%P	Exploratory
19	Flow Cytometry	Activated monocytes	AI	CD3-CD19-/CD14+HLADR+,%P	Exploratory
20	Flow Cytometry	Inflammatory monocytes	AJ	CD3-CD19-/Q2: CD16+, CD14+,%P	Exploratory
21	Flow Cytometry	B cells	AK	CD19+,%P	Exploratory
22	Flow Cytometry	NK cells	AL	CD56+,%P	Exploratory
23	Flow Cytometry	Non CD3 NK cells	AM	cells/singlets/DUMP-/cd56+cd3- cells,Freq. of Parent	Exploratory
24	Flow Cytometry	NKT cells	AN	CD56+CD3+,%P	Exploratory
25	Flow Cytometry	Bright NK cells CD16+	AO	CD56+CD3-/CD56++CD16+,%P	Exploratory
26	Flow Cytometry	Bright NK cells CD16-	AP	CD56+CD3-/CD56++CD16-,%P	Exploratory
27	Flow Cytometry	CD16+ NK cells	AQ	CD56+CD3-/Q2: CD56+,CD16+,%P	Exploratory
28	Flow Cytometry	CD16- NK cells	AR	CD56+CD3-/Q3: CD56+, CD16-,%P	Exploratory
29	Flow Cytometry	CD4+CD26+	AS	CD4+CD26+ total,%P	Exploratory
30	Flow Cytometry	CD4+CD27+	AT	CD4+CD27+ total,%P	Exploratory
31	Flow Cytometry	CD4+CD161+	AU	CD4+CD161+ total,%P	Exploratory
32	Flow Cytometry	CD4 MAITS	AV	CD4+Va7.2+,%P	Exploratory
33	Flow Cytometry	CD4 MAITS CD27+	AW	CD4+Va7.2+CD27+,%G	Exploratory
34	Flow Cytometry	CD4 MAITS CD26+	AX	CD4/Q26: CD26+Va7-2+,%P	Exploratory
35	Flow Cytometry	CD4 MAITS CD161+	AY	CD4/Q30: CD161+Va7-2+,%P	Exploratory
36	Flow Cytometry	CD8+CD26+	AZ	CD8+CD26+total,%P	Exploratory

37	Flow Cytometry	CD8+CD27+	BA	CD8+CD27+total,%P	Exploratory
38	Flow Cytometry	CD8+CD161+	BB	CD8+CD161+total,%P	Exploratory
39	Flow Cytometry	CD8 MAITS	BC	CD8+Va7.2+ total,%P	Exploratory
40	Flow Cytometry	CD8 MAITS CD27+	BD	CD8+Va7.2+CD27+,%G	Exploratory
41	Flow Cytometry	CD8 MAITS CD26+	BE	CD8/Q14: CD26+Va7-2+,%P	Exploratory
42	Flow Cytometry	CD8 MAITS CD161+	BF	CD8/Q30: CD161+Va7-2+,%P	Exploratory
43	Flow Cytometry	CD163+ Monocytes (anti-Inflammatory)	BG	CD14-CD163 total	Exploratory
44	Flow Cytometry	Transitional-Regulatory B cells	BH	Cells/singlets/singlets2/time/live cells/cd19+/CD24++CD38++(Transit-Reg?),%P	Exploratory
45	Flow Cytometry	Plasmoblasts1	BI	Cells/singlets/singlets2/time/live cells/cd19+/CD27+CD38+(PB),%P	Exploratory
46	Flow Cytometry	Plasmoblasts2	BJ	Cells/singlets/singlets2/time/live cells/cd19+/CD38+CD24-(PB),%P	Exploratory
47	Flow Cytometry	lasmoblasts3	BKP	Cells/singlets/singlets2/time/live cells/cd19+/IgD-CD27++(PB),%P	Exploratory
48	Flow Cytometry	Immature B cells	BL	Cells/singlets/singlets2/time/live cells/cd19+/IgD-CD27-/CD24++CD38++(immature),%P	Exploratory
49	Flow Cytometry	IgG+CD5+ B cells	BM	Cells/singlets/singlets2/time/live cells/cd19+/Q14: IgG+,, CD5+,%P	Exploratory
50	Flow Cytometry	Memory B cells	BN	Cells/singlets/singlets2/time/live cells/cd19+/Q37: IgDÑ,, CD27+,%P	Exploratory
51	Flow Cytometry	Naiive B cells	BO	Cells/singlets/singlets2/time/live cells/cd19+/Q39: IgD+,, CD27Ñ(Naiive),%P	Exploratory
52	Flow Cytometry	CD5+ B cells	BP	Cells/singlets/singlets2/time/live cells/cd19+/total CD5,%P	Exploratory
53	Flow Cytometry	CD24+ B cells	BQ	Cells/singlets/singlets2/time/live cells/cd19+/total CD24,%P	Exploratory
54	Flow Cytometry	CD27+ B cells	BR	Cells/singlets/singlets2/time/live cells/cd19+/total CD27,%P	Exploratory
55	Flow Cytometry	CD38+ B cells	BS	Cells/singlets/singlets2/time/live cells/cd19+/total CD38,%P	Exploratory

56	Flow Cytometry	IgD+ B cells	BT	Cells/singlets/singlets2/time/live cells/cd19+/total IgD,%P	Exploratory
57	Flow Cytometry	IgG+ B cells	BU	Cells/singlets/singlets2/time/live cells/cd19+/total IgG,%P	Exploratory
58	Flow Cytometry	Inhibitory CD4 cells	BV	Total CD4 PD1	Exploratory
59	Flow Cytometry	Inhibitory CD8 cells	BW	Total CD8 PD1	Exploratory
60	Flow Cytometry	Monocytes	BX	Total CD14	Exploratory
61	Flow Cytometry	Monocyte	BY	ML_Ratio	Exploratory
62	Flow Cytometry	Gamma delta cells	BZ	cells/singlets/DUMP-/CD3/CD4-CD8-,Freq. of Parent	Exploratory
63	Multiplex Assay (Luminex)	sCD40L	CA	Plasma samples	Exploratory
64	Multiplex Assay (Luminex)	EGF	CB	Plasma samples	Exploratory
65	Multiplex Assay (Luminex)	Eotaxin/CCL11	CC	Plasma samples	Exploratory
66	Multiplex Assay (Luminex)	FGF-2	CD	Plasma samples	Exploratory
67	Multiplex Assay (Luminex)	Flt-3 ligand	CE	Plasma samples	Exploratory
68	Multiplex Assay (Luminex)	Fractalkine	CF	Plasma samples	Exploratory
69	Multiplex Assay (Luminex)	G-CSF	CG	Plasma samples	Exploratory
70	Multiplex Assay (Luminex)	GM-CSF	CH	Plasma samples	Exploratory
71	Multiplex Assay (Luminex)	GRO	CI	Plasma samples	Exploratory

72	Multiplex Assay (Luminex)	IFN-α2	CJ	Plasma samples	Exploratory
73	Multiplex Assay (Luminex)	IFN-γ	CK	Plasma samples	Exploratory
74	Multiplex Assay (Luminex)	IL-1α	CL	Plasma samples	Exploratory
75	Multiplex Assay (Luminex)	IL-1β	CM	Plasma samples	Exploratory
76	Multiplex Assay (Luminex)	IL-1ra	CN	Plasma samples	Exploratory
77	Multiplex Assay (Luminex)	IL-2	CO	Plasma samples	Exploratory
78	Multiplex Assay (Luminex)	IL-3	CP	Plasma samples	Exploratory
79	Multiplex Assay (Luminex)	IL-4	CQ	Plasma samples	Exploratory
80	Multiplex Assay (Luminex)	IL-5	CR	Plasma samples	Exploratory
81	Multiplex Assay (Luminex)	IL-6	CS	Plasma samples	Exploratory
82	Multiplex Assay (Luminex)	IL-7	CT	Plasma samples	Exploratory
83	Multiplex Assay (Luminex)	IL-8	CU	Plasma samples	Exploratory
84	Multiplex Assay (Luminex)	IL-9	CV	Plasma samples	Exploratory

85	Multiplex Assay (Luminex)	IL-10	CW	Plasma samples	Exploratory
86	Multiplex Assay (Luminex)	IL-12 (p40)	CX	Plasma samples	Exploratory
87	Multiplex Assay (Luminex)	IL-12 (p70)	CY	Plasma samples	Exploratory
88	Multiplex Assay (Luminex)	IL-13	CZ	Plasma samples	Exploratory
89	Multiplex Assay (Luminex)	IL-15	DA	Plasma samples	Exploratory
90	Multiplex Assay (Luminex)	IL-17A	DB	Plasma samples	Exploratory
91	Multiplex Assay (Luminex)	IP-10	DC	Plasma samples	Exploratory
92	Multiplex Assay (Luminex)	MCP-1	DD	Plasma samples	Exploratory
93	Multiplex Assay (Luminex)	MCP-3	DE	Plasma samples	Exploratory
94	Multiplex Assay (Luminex)	MDC (CCL22)	DF	Plasma samples	Exploratory
95	Multiplex Assay (Luminex)	MIP-1α	DG	Plasma samples	Exploratory
96	Multiplex Assay (Luminex)	MIP-1β	DH	Plasma samples	Exploratory
97	Multiplex Assay (Luminex)	TGF-α	DI	Plasma samples	Exploratory

98	Multiplex Assay (Luminex)	TNF-α	DJ	Plasma samples	Exploratory
99	Multiplex Assay (Luminex)	TNF-β	DK	Plasma samples	Exploratory
100	Multiplex Assay (Luminex)	VEGF	DL	Plasma samples	Exploratory
101	Flow Cytometry	CD3+CD161+	DM	cd3cd161	Exploratory
102	Flow Cytometry	CD161+MAITS	DN	cd3cd161/VA7.2+CD161+,Freq. of Grandparent	Exploratory
103	Flow Cytometry	CD3+CD26+	DO	cd3+cd14-/CD26,Freq. of Parent	Exploratory
104	Flow Cytometry	CD3+CD27+	DP	cd3+cd14-/cd27+,Freq. of Parent	Exploratory
105	Flow Cytometry	CD3+ MAITS	DQ	cells/singlets/singlets2/time/Dump-cd3+/cd3+cd14-/cd3va7.2,Freq. of Parent	Exploratory

2.4 Sample size considerations

The number of assays selected was determined by practical limiting factors of the sample volume, laboratory capacity and study budget. No formal sample size considerations were taken in the design of this experiment.

2.5 Assay methodology and blinding

The scientists performing all assays in conjunction with the case-control study will be blinded as to treatment group (MVA85A or control) and as to whether a sample is from a case or a matched control. All assays will be performed according to relevant Oxford SOPs. Donor samples, once drawn, will be assigned to one of 7 processing runs. Samples were collected from 3 study sites over a period of 3 years during the Phase IIb study. Sample sets from the different study sites will be distributed evenly across the 7 processing runs and randomized by time of recruitment into the study. Randomization of samples will be implemented based on a sequence of random numbers which will be used to generate 7 batches of samples for processing.

Details on individual assays are included in Appendix 2.

3 STATISTICAL METHODS

The following is an overview of the statistical methods to be performed in conjunction with the immune correlates analysis for Study C-020-485.

Analyses performed on all assays/variables in Table 1:
Preliminary data review: univariate descriptive statistics, boxplots to identify outliers for all selected assays/antigens; correlations review and preliminary assessment of potential multicollinearity considerations
Selection of primary variables: receiver operating characteristic (ROC) curve and conditional logistic regression, using all variables within a given assay. Selection of key independent variables across all assays for inclusion in primary model, with maximum number of variables selected for inclusion in the model based on power considerations and using the approach specified below.

Analyses performed on select assays/variables (following initial reviews and down selection):
Analysis of primary objectives: conditional logistic regression model using subset of three key variables as defined in the primary objectives above, to address primary objectives.
Analysis of exploratory objective: additional logistic regression models using other assay response variables as well as these three key variables.
Review of model fit using bootstrapping.:

3.1 Preliminary data review

Given the potential impact of data outliers on summarization of variability and model assumptions, a careful review of outliers will be undertaken for all variables in the selected assays in Table 1. A review of immune response variables, including any positive and negative controls for each subject/assay, as available, will be facilitated using graphical displays (e.g., box plots) and univariate descriptive statistics (e.g., mean, median, standard deviation, and minimum and maximum values) for all variables at Day -7. Summaries will be presented over all subjects, initially pooled over cases and controls (n=172), with additional summaries stratified by treatment group.

Cell based assays will be performed only on samples where the viability of cells is >50% (see Appendix 3). For each infant, the percentage of live cells on which cell based assay results are generated will be determined and assay results will be excluded from analysis if cell viability is less than or equal to 50%. For certain analyses such as conditional logistic regression, this will mean matched control subjects will be excluded if the case results are excluded.

Outliers will be queried to Oxford for clarification of response. Outliers will be identified via a review of the distribution of the variables. Tukey's Rule[1], which provides a distribution free method for identification of outliers, will be used to set upper and lower limits for preliminary review and identification of outliers. After determining the 25th and 75th percentiles, the upper limit is set as P75 + 3 times the interquartile range, or P75-P25. Likewise, the lower limit is P25 – 3(P75-P25). Following this review, only outliers which are identified and determined to be the result of contamination, operator error, or mechanical failure will be excluded from the primary analyses after confirmation by Oxford. These outliers will be excluded from the determination of the primary analysis. Additional sensitivity analyses will be performed if the proportion of samples excluded following this review exceeds 10% or based on observed missing patterns in available data. Correlations and dependencies within each assay will be examined using Spearman's rank correlations.

Data distributions will be reviewed and appropriate transformations considered This review will be conducted within each assay (all variables within a given assays), and across assays (all variables), as appropriate. Based on initial reviews, transformation options (e.g., power transformations, likelihood functions using Box-Cox) will be considered and transformed independent variables will be examined for later inclusion in the conditional logistic regression models. Outputs from these reviews will include histograms and scatter plots which will help assess transformations and next steps.

All analyses will be performed based on data received; no imputation of missing values will be performed prior to statistical analysis, and it is assumed/to be confirmed that any missing data

[1] Tukey, J. W. (1977). *Exploratory data analysis.* Reading, MA: Addison-Wesley Publishing Company.

will be missing at random. Documentation of missing data will be submitted with the final experimental report.

3.2 Variables to be included in the model to answer the primary objective

This model is pre-specified, based on the results of The 020 TB disease correlate analysis (Fletcher H et al, Nature Comms 2016), to include Activated CD4+ T cells, BCG-Specific Elispot responses and Ag85A IgG, to predict occurrence of *M.tb* infection. The model will include a variable indicating the controls who are matched to each case. In a small number of cases (around 7%) day 28 data will be used where day-7 data is missing. This should have very little/no effect on the results as no efficacy post vaccination was detected, however this will be adjusted for in the model, along with whether each person was randomised to receive MVA85A or not in the original trial.

3.3 Selection of variables to be included in the model to answer the secondary objective

Following adjudication of outliers in the data review as described in Section 3.1, a core set of primary assays and corresponding secondary immune response variables will then be identified for inclusion in the subsequent model, as follows.

All immune response variables in each of the assays in Table 1 will be initially considered for selection as primary variables. Selection will be based on (a) immunological review and (b) statistical review, as follows:

> **Immunological review:** Preliminary identification of key immune response variables for each assay based on anticipated immune response. A list of primary variables from amongst all variables in each of the Table 1 assays will be identified by Oxford Immunology, based on criteria identified by the Oxford immunology working group for C-020-485 (under separate cover). These key variables will be considered for the primary model pending a separate statistical review as described below. Variables not ultimately selected will be considered for inclusion in sensitivity analyses (exploratory objective).
>
> **Statistical review:** Preliminary identification of key immune response variables based on correlations. Conduct preliminary ROC and conditional logistic regression (odds ratio) for each assay/antigen, identify set of primary variables.
>
> A statistical evaluation of each assay/antigen will be undertaken, based on values recorded at Study Day -7 (Pre-vaccination, all subjects), including treatment status). All variables listed in Table 1 will be considered, as follows:

3.3.1 Univariate analysis

For each assay represented in Table 1 which has been identified as a key immune response variable in the immunological review, a series of univariate conditional logistic regression analyses will be performed to assess the association between Day -7 immune response and risk of *M.TB* infection. In these analyses, the occurrence of *M.tb* infection will be the outcome variable with the matched case-control set number as the strata variable and the Day -7 immune response as the independent variable. Results will be summarized as a list of all immune response variables together with their estimated odds ratios, 95% confidence intervals, and two-sided p-values. Q values (which correct for multiple comparisons by estimating the false discovery rate using the Benjamini and Hochberg method) will be presented. Q values less than 0.20 will be considered as evidence of a significant association. Also, the area under the receiver operating characteristic (AUROC) curve will be calculated for each immune response. Immune responses with AUROC between 0.80 and 0.90 will be considered as good discriminators of high and low risk infants while responses with AUROC greater than 0.90 will be considered excellent discriminators.

3.3.2 Multivariate analysis

All Day -7 immune responses found to be significantly associated with risk of *M.tb* infection (q value ≤ 0.20) or with an AUROC of at least 0.80 will be considered for inclusion in a multivariate conditional logistic regression model. However, immune responses which are strongly correlated with another immune response (absolute Spearman's rank correlation coefficient ≥ 0.60) will be excluded. When such collinearity exists, the immune response with the higher degree of missing data will be dropped. From the remaining set of uncorrelated immune responses, a stepwise conditional logistic regression analysis will be performed. At each step, the estimated odds ratio for each immune response in the model will be calculated along with the Akaike information criterion (AIC) and the AUROC based on the linear combination of the immune responses. The best model will be based on the AIC and the relative improvement to the AUROC. The internal validity of the best model will be assessed by obtaining a bootstrapped estimate of the AUROC based on 500 samples with replacement of the matched case-control set numbers included in the model.

The final model will be adjusted, if appropriate, for whether D-7 or D28 data was used and whether the participant was randomised to MVA85A in the original trial.

All regression assumptions will be checked, and a review of the distribution of residuals will be performed graphically after the completion of the final model, above. The Pearson and deviance chi-square goodness-of-fit statistics will be examined to explore the assumptions of the goodness-of-fit statistics. In particular, concerns around multicollinearity (interactions among the assay immune response variables) will be examined throughout the modeling process as described in Section 3.3.

The model fitting portion of each analysis will be repeated, for validation purposes, using receiver

operator characteristic (ROC) analysis and bootstrapping techniques to identify classification accuracy. The performance of a model will be evaluated based on the area under the ROC curve, which presents sensitivity and 1-specificity (the false positive rate). Bootstrapping resampling will then be used to evaluate model fit as assessed by area under the ROC curve. The distribution of the areas under the ROC curves and associated 95% CIs will be used to summarize the classification accuracy of the models.

4 Computer methods

Statistical analyses will be performed using Stata v15.

5 Validation and Data Storage

Results of all statistical analyses as outlined above will be reviewed by a second, independent statistician, prior to dissemination of results.

Following finalization of a summary report for all findings, results of all analyses, including all relevant supportive derived datasets and programs, will be stored in a secure location at Oxford University, as determined by the PI, Prof McShane.

Appendixes:

Appendix 1: Summary of matched control selections

Three controls were matched to each individual *M.tb* infection case. This matching was based on protocol-specified definition and methodologies documented elsewhere. N=43 subjects have been identified as meeting case definitions *M.tb* infection**.**

Eligible Controls dataset

A dataset containing all Study Group 5 (Correlate of Protection Cohort) subjects in the Safety population (n=2287) was initially created.

Following team reviews and discussions, the final dataset excludes Cohort 5 subjects who meet any of the following criteria:

(a) meet Endpoint #1, #2 or #3 case definition;
(b) death (n=10);
(c) heelstick subject (n=100);
(d) received TB treatment or prophylaxis at any point during the study (n=231 of 533 subjects)

Based on the criteria above, the Eligible Controls dataset is restricted to a total of 1351 Group 5 subjects.

Impact of matching on analysis:

The selection of controls using matching considerations as identified by the C-020 team will have an impact on the interpretation of the estimate of the odds ratios during the analysis phase, and handling of this must be documented in the SAP. Further, analysis of any impact of time on study or weight, in relation to developing *M.tb* infection, will be restricted as these variables are included in the matching process.

Validation of datasets: The selection of subjects in the Cases and in the Eligible Controls datasets has been validated by an independent programmer, prior to the use of these datasets in the matching controls selection process.

Summary of Matching Requirements for Cases and Controls

**Variable**	**Cases (n=43)**	**Controls (n=129)**	**P value**
Gender (Male) (n %)	27 (63)	67 (52)	0.2

Race (Coloured) (n %)	29 (67)	87 (67)	1
Enrolment Age (Mean, SD)	4.8 (0.47)	4.8 (0.43)	0.9
Time on Study (Mean, SD)	25.2 (5.8)	25 (5.6)	0.8
Weight Centile (Mean, SD)	39 (26.5)	38.5(26.5)	0.9

	Case	Control
Black	14	42
Coloured	29	87
Female	16	62
Male	27	67

Appendix 2: Summary of C-020-485 immune correlates assays

IFN-γ ELISPOT assay for the detection of an antigen-specific immune responses

Antigen specific immune responses were detected in fresh PBMC from the TB020 trial using the IFN-γ ELISPOT assay and the WB-ICS assay but we were not able to detect a response when using a frozen PBMC ICS assay [2].

In the correlates of risk of TB disease in infants, we have reported a negative association between BCG-specific T cells secreting IFN-γ and risk of TB disease [3]. In previous work we have shown a correlation between the ELISPOT, WB-ICS and PBMC-ICS assays [5]indicating that although sensitivity is variable for the measurement of strong antigen specific IFN-γ immune response these assays are similar. Given that 1) there are only frozen PBMC (not fresh PBMC or whole blood) available for the immune correlates analysis 2) the ELISPOT assay is sensitive enough to detect very low numbers of antigen specific immune cells and the immune responses to MVA85A in this population are modest 3) the ELISPOT assay uses less cells than PBMC-ICS and 4) the ELISPOT assay has been used throughout the development of MVA85A to measure the magnitude of the antigen specific immune response the ELISPOT was selected for inclusion in the immune correlates analysis.

For the ELISPOT assay we tested further parameters including the use of duplicate wells versus triplicate wells. Inter assay reliability was tested for duplicate and triplicate wells and reliability was found to be very high ICC >0.9 with no difference in reliability for duplicates versus triplicates. However, the IFN-γ ELISPOT responses to BCG and PPD were significantly higher (P<0.01) when cells were rested for 2 hours after thawing compared to resting overnight. As relatively low numbers of cells are required for the ELISPOT assay we will also able to assess the ability of the

infants to mount a CD8+ T cell response (CD8 epitopes for CMV and EBV) and will be able to assess the immune response to BCG.

Readouts for the ELISPOT assay will be the number of IFN-γ producing spot forming cells per million PBMC for each antigen tested.

Figure 1. ELISPOT responses observed in pilot studies using samples from infants in the TB020 trial

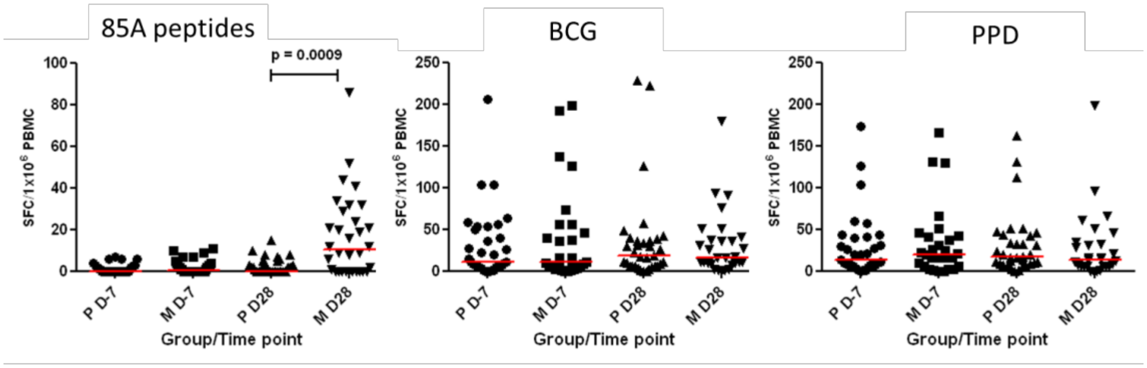

P=placebo
M=MVA85A

In pilot studies antigen specific immune responses to BCG, PPD and Ag85A peptides were modest but detected in most infants (Figure 1).

Gene expression analysis (GEX)

There is a separate SAP for the analysis of transcriptomics data

Mycobacterial growth inhibition assay (MGIA)

Mycobacteria added to PBMC rapidly undergo phagocytosis and thus become subject to cellular immune mechanisms, both innate and vaccine induced. Overall assessment of these antimycobacterial mechanisms can be accomplished by comparing mycobacterial viability (CFU) of completed culture to that of the inoculum. In this study we will be using an optimized MGIA method using autologous serum, this protocol was not developed when the previous study of correlates of risk of TB disease was done. In this method, time to positivity (TTP) in mycobacterial growth indicator tubes (MGIT) is used instead of conventional colony counts as the readout. This has the advantage of greater sensitivity and reproducibility as well as reduced technician time and exposure hazard [6]. MGIT TTP is inversely proportional to the log of the inoculum size – the

larger the number of inoculating mycobacteria, the shorter the TTP. This relationship is utilized to quantify the change in mycobacterial viability during culture. Using a standard curve enables conversion of time to positivity (TTP) of a sample tube into an initial mycobacterial inoculum volume. This inoculum volume is then converted to CFU/ml (experimentally determined for each bacterial stock). The CFU count of each sample tube is divided by the CFU count of the growth control tube, divided by 4 days and finally log converted. This gives the delta log growth per day for each sample tube.

MGIT = Mycobacterial Growth Indicator Tube
CFU = Colony Forming Unit (a measure of mycobacterial viability)
TTP = Time to Positivity (time taken to reach a predetermined level of fluorescence as a measure of growth)

Figure 2 Mycobacterial growth inhibition observed using samples from infants in the TB020 trial

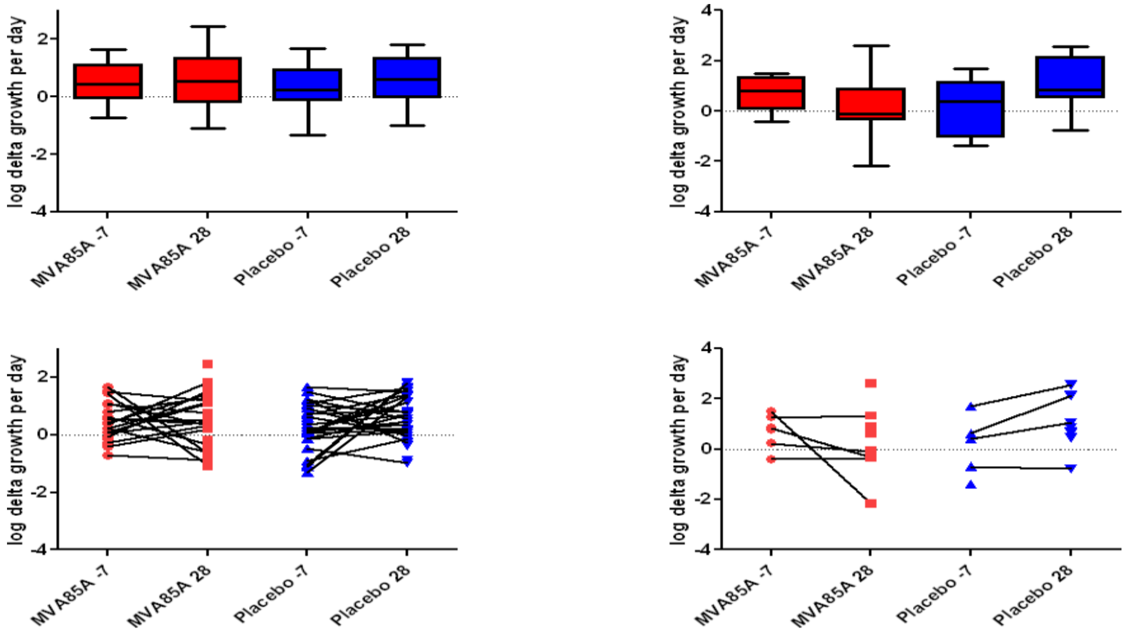

In pilot studies we did not see a significant difference in growth inhibition between the groups (Figure 2: box plots and line plots of the same data).

Flow surface staining

Cell surface flow cytometry will be used to phenotype immune cells in the frozen PBMC of infants

in the TB020 trial. In the previous correlates of risk of TB disease, we reported an association between activated HLA-DR+, CD4+ T cells and increased risk of TB disease [3]. There is some evidence that the proportions of monocytes and lymphocytes in peripheral blood may be correlated with both risk of disease and vaccine efficacy (Fletcher *et al* (in preparation), [7] [8] [9]). We have also found that regulatory T cell markers are associated with MVA85A vaccine immunogenicity [10-13]. The cell populations that will be included in the immune correlates analysis are in Table 1 above. Data on relative proportions of leukocyte subsets can also be used to deconvolute gene expression signatures. This may allow distinction of gene expression differences that reflect changes in blood cell subsets (e.g. changes in relative proportions of monocytes and lymphocytes) from gene expression patterns that are independent of leukocyte subset proportions (e.g. changes in phenotypic, activation or functional markers).

Enzyme-linked Immunosorbent Assay (ELISA)

We have reported that plasma levels of Ag85A-specific IgG were negatively associated with risk of TB disease [3]. In this study levels of IgG will be measured in plasma samples collected at D-7 and D28 of the study. Background subtracted OD values will be presented.

Multiplex Assay (Luminex):

Luminex assay will be done on D-7 plasma samples using cytokine and chemokines Multiplex assay looking at a total of 41 analytes.

Appendix 3: Data Handling

Cell Viability

Data will be excluded from infant samples where cell viability is < 50% (<50% live cells in total cell population as measured using Vivid live/dead stain and flow cytometry). Where cell viability data as measured by flow cytometry is not available the IFN-ψ ELISPOT response to PHA will be used as a measure of cell functionality. Samples with no flow cytometry data and an IFN-ψ ELISPOT response to PHA >1000 SFC/million PBMC will be included in the analysis.

ELISPOT

Spot forming cells were counted using an automated ELISPOT reader. Each well contained 300,000 cells and each sample was plated in duplicate. The mean value for duplicate wells was calculated and divided by 0.3 to obtain the number of spot forming cells (SFC) per million PBMC. The SFC from the unstimulated control well was subtracted from the sample well. Sample values less than 0 were adjusted to 0. Data for this analysis was provided as background subtracted SFC/million PBMC.

MGIA

MGIA data was reported as time to positivity (TTP) using a Bactec MGIT 960 liquid culture machine. Duplicate tubes were run for each sample and the mean value of duplicate tubes used in the analysis. Samples were excluded from analysis where the standard deviation between replicate tubes was >40%. TTP values were converted to log CFU and log CFU of the growth control tube was subtracted from log CFU of each sample tube as previously described [6]. Data for this analysis was provided as log CFU of sample tube - log CFU of growth control tube.

Flow cytometry

Gating strategy is described in flow SOP. Flow data is reported as frequency of live cells with the exception of activated cells and regulatory T cells which are frequency of parent.

ELISA

Optical density values of Ag85A and BCG-specific IgG will be presented. Mean OD values of duplicate wells will be calculated and mean OD values of blank wells will be subtracted. OD values less than 0 will be adjusted to 0.

Multiplex Assay (Luminex):

Concentrations of 41 cytokines and chemokines will be measured in plasma samples collected at day -7.

*these assays form the primary analysis

References:

1. Hatherill, M., S. Verver, and H. Mahomed, *Consensus statement on diagnostic end points for infant tuberculosis vaccine trials.* Clin Infect Dis, 2012. **54**(4): p. 493-501.
2. Tameris, M.D., et al., *Safety and efficacy of MVA85A, a new tuberculosis vaccine, in infants previously vaccinated with BCG: a randomised, placebo-controlled phase 2b trial.* Lancet, 2013. **381**(9871): p. 1021-8.
3. Fletcher, H.A., et al., *T-cell activation is an immune correlate of risk in BCG vaccinated infants.* Nat Commun, 2016. **7**: p. 11290.
4. Harris, S.A., et al., *Process of assay selection and optimization for the study of case and control samples from a phase IIb efficacy trial of a candidate tuberculosis vaccine, MVA85A.* Clin Vaccine Immunol, 2014. **21**(7): p. 1005-11.
5. Beveridge, N.E., et al., *A comparison of IFNgamma detection methods used in tuberculosis vaccine trials.* Tuberculosis (Edinb), 2008. **88**(6): p. 631-40.

6. Fletcher, H.A., et al., *Inhibition of mycobacterial growth in vitro following primary but not secondary vaccination with Mycobacterium bovis BCG.* Clin Vaccine Immunol, 2013. **20**(11): p. 1683-9.
7. Warimwe, G.M., et al., *The ratio of monocytes to lymphocytes in peripheral blood correlates with increased susceptibility to clinical malaria in Kenyan children.* PLoS One, 2013. **8**(2): p. e57320.
8. Warimwe, G.M., et al., *Peripheral blood monocyte-to-lymphocyte ratio at study enrollment predicts efficacy of the RTS,S malaria vaccine: analysis of pooled phase II clinical trial data.* BMC Med, 2013. **11**: p. 184.
9. Naranbhai, V., et al., *Ratio of monocytes to lymphocytes in peripheral blood identifies adults at risk of incident tuberculosis among HIV-infected adults initiating antiretroviral therapy.* J Infect Dis, 2014. **209**(4): p. 500-9.
10. Fletcher, H.A., et al., *Boosting BCG vaccination with MVA85A down-regulates the immunoregulatory cytokine TGF-beta1.* Vaccine, 2008. **26**(41): p. 5269-75.
11. de Cassan, S.C., et al., *Investigating the induction of vaccine-induced Th17 and regulatory T cells in healthy, Mycobacterium bovis BCG-immunized adults vaccinated with a new tuberculosis vaccine, MVA85A.* Clin Vaccine Immunol, 2010. **17**(7): p. 1066-73.
12. Griffiths, K.L., et al., *Th1/Th17 cell induction and corresponding reduction in ATP consumption following vaccination with the novel Mycobacterium tuberculosis vaccine MVA85A.* PLoS One, 2011. **6**(8): p. e23463.
13. Matsumiya, M., et al., *Roles for Treg expansion and HMGB1 signaling through the TLR1-2-6 axis in determining the magnitude of the antigen-specific immune response to MVA85A.* PLoS One, 2013. **8**(7): p. e67922.
