# Supplementary material for: Inflammation and immune activation are associated with risk of Mycobacterium tuberculosis infection in BCG-vaccinated infants
Source: Nat Commun. 2022 Nov 3;13:6594. doi: 10.1038/s41467-022-34061-7 (PMC9632577; doi:10.1038/s41467-022-34061-7)
Supplement: Supplementary file 3 — Description of Additional Supplementary Files [file 41467_2022_34061_MOESM3_ESM.pdf]

Description of Additional Supplementary Files

Supplementary Data 1: Baseline characteristics of participant by *M.tb* infection.

Supplementary Data 2: Univariate conditional logistic regression and AUC analysis results. Two-sided *p*-values were reported. *P*-values adjusted by Benjamini-Hochberg multiple testing correction were also reported.

Supplementary Data 3: Univariate logistic regression results (*M.tb*-infected vs *M.tb*-uninfected infants) of plasma levels of cytokines, chemokines and complements. Two-sided *p*-values were reported. Only *p*-values of the plasma levels of cytokines, chemokines and complements were used for adjusting Benjamini-Hochberg multiple testing correction.

Supplementary Data 4:

a: The influence of time points of samples on immune parameters. Two-sided Mann-Whitney test was used for comparison and Benjamini-Hochberg multiple testing correction was used.

b: The influence of the time points of samples on the results of conditional logistic regression. The 2nd and 4th column are odds ratio and *p*-values when we use only Day -7 samples for conditional logistic regression, the 3rd and 5th column are odds ratio and *p*-values when we use both Day -7 and Day 28 samples for conditional logistic regression. The 6th column is the ratio of odds ratio when we used both Day -7 and Day 28 samples and that when we use only Day -7 samples. Two-sided *P*-values were not adjusted for multiple testing correction.

Supplementary Data 5:

a: Difference of immune parameters between CMV-infected and CMV-uninfected infants. Two-sided Mann-Whitney test was used for comparison. *P*-values adjusted by Benjamini-Hochberg multiple testing correction were also reported.

b: Difference of immune parameters measured by multiplex assays between CMV-infected and CMV-uninfected infants. Two-sided Mann-Whitney test was used for comparison. *P*-values were adjusted only within these immune parameters by Benjamini-Hochberg multiple testing correction.

Supplementary Data 6:

a: Differentially expressed genes between *M.tb*-infected vs *M.tb*-uninfected infants among all infants.

b: Differentially expressed genes between *M.tb*-infected and *M.tb*-uninfected infants among CMV-infected infants.

c: Differentially expressed genes between *M.tb*-infected vs *M.tb*-uninfected infants among CMV-uninfected infants.

d: Differentially expressed genes between CMV-infected vs CMV-uninfected infants.

Two-sided Wald test was used to calculate *P*-values. *P*-values from the subset of genes that passed the independent filtering step were adjusted using Benjamini-Hochberg multiple testing correction.

Supplementary Data 7:

a: Upregulated genes in enriched gene sets. CERNO algorithm and gene sets defined by Li *et al.* [1] were used in gene set enrichment.

b: Downregulated genes in enriched gene sets. CERNO algorithm and gene sets defined by Li *et al.* [1] were used in gene set enrichment.

Supplementary Data 8:

Gene sets enrichment results using overrepresentation test and gene sets defined by GO database. One-sided *P*-values were adjusted by Benjamini-Hochberg multiple testing correction.

Supplementary Data 9: Enriched gene sets and corresponding differentially expressed genes for differential expression between TB progressor and non-progressor in adolescents in the ACS. Gene sets enrichment results were acquired by the CERNO algorithm and gene sets were defined by Li *et al* [1].

a: Upregulated genes in enriched gene sets.

b: Downregulated genes in enriched gene sets.

Supplementary Data 10:

a: Cellular and humoral assays performed on samples from study subjects.

b: Percentages of samples that had all planned assays performed, based on cells availability for the assays.

Supplementary Data 11: Flow cytometry panels to characterise (1) T, (2) B and (3) MAIT-cells populations.

Supplementary Data 12: Immunology data.

Supplementary Data Reference

1. Li, S. *et al.* Molecular signatures of antibody responses derived from a systems biology study of five human vaccines. *Nat. Immunol.* **15**, 195–204 (2014).
