# Supplementary material for: Inflammation and immune activation are associated with risk of Mycobacterium tuberculosis infection in BCG-vaccinated infants
Source: Nat Commun. 2022 Nov 3;13:6594. doi: 10.1038/s41467-022-34061-7 (PMC9632577; doi:10.1038/s41467-022-34061-7)
Supplement: Supplementary file 16 — Reporting Summary [file 41467_2022_34061_MOESM16_ESM.pdf]

nature portfolio

Corresponding author(s): Helen McShane

Last updated by author(s): Oct 25, 2022

Reporting Summary

Nature Portfolio wishes to improve the reproducibility of the work that we publish. This form provides structure for consistency and transparency in reporting. For further information on Nature Portfolio policies, see our Editorial Policies and the Editorial Policy Checklist.

Statistics

For all statistical analyses, confirm that the following items are present in the figure legend, table legend, main text, or Methods section.

n/a	Confirmed	
☐	☒	The exact sample size (n) for each experimental group/condition, given as a discrete number and unit of measurement
☐	☒	A statement on whether measurements were taken from distinct samples or whether the same sample was measured repeatedly
☐	☒	The statistical test(s) used AND whether they are one- or two-sided<br>*Only common tests should be described solely by name; describe more complex techniques in the Methods section.*
☐	☒	A description of all covariates tested
☐	☒	A description of any assumptions or corrections, such as tests of normality and adjustment for multiple comparisons
☐	☒	A full description of the statistical parameters including central tendency (e.g. means) or other basic estimates (e.g. regression coefficient) AND variation (e.g. standard deviation) or associated estimates of uncertainty (e.g. confidence intervals)
☐	☒	For null hypothesis testing, the test statistic (e.g. F, t, r) with confidence intervals, effect sizes, degrees of freedom and P value noted<br>*Give P values as exact values whenever suitable.*
☒	☐	For Bayesian analysis, information on the choice of priors and Markov chain Monte Carlo settings
☒	☐	For hierarchical and complex designs, identification of the appropriate level for tests and full reporting of outcomes
☐	☒	Estimates of effect sizes (e.g. Cohen's d, Pearson's r), indicating how they were calculated

Our web collection on statistics for biologists contains articles on many of the points above.

Software and code

Policy information about availability of computer code

Data collection	Gen5 (v2.07)<br>BD FACSDiva (v6.2)<br>AID (v7.0 iSpot)<br>xPONENT (v4.2.1705.0)

Data analysis	Flowjo (v8.8)<br>Stata (v14)<br>R (v4.1.2)<br>HISAT2 (v2.1.0)<br>featureCounts (subread v2.0.2)<br>fastqc (v0.11.8)<br>DESeq2 (v1.22.2)<br>tmod (v0.40)<br>clusterProfiler (v 3.10.1)<br>GOfuncR (v1.2.0)<br>drLumi (v0.1.2)<br>simplifyEnrichment (v1.4.0)<br>GOSemSim (2.16.1)<br>CERNO algorithm (tmod v0.40)

For manuscripts utilizing custom algorithms or software that are central to the research but not yet described in published literature, software must be made available to editors and reviewers. We strongly encourage code deposition in a community repository (e.g. GitHub). See the Nature Portfolio guidelines for submitting code & software for further information.

Data

Policy information about availability of data

All manuscripts must include a data availability statement. This statement should provide the following information, where applicable:

- Accession codes, unique identifiers, or web links for publicly available datasets
- A description of any restrictions on data availability
- For clinical datasets or third party data, please ensure that the statement adheres to our policy

The RNA-Seq datasets generated during in this study have been deposited in the Gene Expression Omnibus database under accession code GSE203395 [https://www.ncbi.nlm.nih.gov/geo/query/acc.cgi?acc=GSE203395].
The transcriptomic data in ACS used in this study are available in the Gene Expression Omnibus database under accession code GSE79362 [https://www.ncbi.nlm.nih.gov/geo/query/acc.cgi?acc=GSE79362].
Source data are provided with this paper.
The immunology datasets generated in this study are provided in the Supplementary Data 12.

Human research participants

Policy information about studies involving human research participants and Sex and Gender in Research.

Reporting on sex and gender	Sex was considered in the study design by choosing matched controls based on sex, ethnic group, Centre for Disease Control (CDC) weight-for-age percentile and time on study so as to remove the influence of sex on our analysis.
Population characteristics	Cohort characteristics are listed in Supplementary Data 1.
Recruitment	Parents/guardians bringing their infants for routine immunizations or other visits at public sector clinics will be asked if they are interested in entering their infant in this research protocol. Study staff may also approach parent/guardians of infants at home using information obtained from clinic records, birth records, word of mouth referrals from community contacts, or after direct contact parents/guardians. They will be informed about the study including the inclusion/exclusion criteria of the study. Written informed consent will be obtained prior to conducting any study-related procedures using a consent form approved by the IRB and signed and dated by the parent/guardian at the time of consent. The clinical investigator, or designee, will conduct the consent discussion on an individual basis with each parent/guardian and will allow adequate time for all questions to be addressed. A copy of the signed consent form will be given to the parent/guardian; the parents/guardians' willingness to continue on the study will also be assessed at each study-related screening visit.<br>In this study, we excluded infants who received IPT, which might include infants who were QFT-positive and did not progress to TB disease but received IPT because they were also TST-positive. For the infants included in our study, we did not know their TST status; it is therefore possible that these infants were not representative of all QFT-positive infants.
Ethics oversight	The trial was approved by the University of Cape Town Faculty of Health Sciences Human Research Ethics Committee, Oxford University Tropical Research Ethics Committee, and the Medicines Control Council of South Africa. Parents or legal guardians provided written, informed consent.

Note that full information on the approval of the study protocol must also be provided in the manuscript.

Field-specific reporting

Please select the one below that is the best fit for your research. If you are not sure, read the appropriate sections before making your selection.

[x] Life sciences [ ] Behavioural & social sciences [ ] Ecological, evolutionary & environmental sciences

For a reference copy of the document with all sections, see nature.com/documents/nr-reporting-summary-flat.pdf

Life sciences study design

All studies must disclose on these points even when the disclosure is negative.

Sample size	All relevant samples available from the study were used.
Data exclusions	Multiplex data that did not pass the QC were excluded.
Replication	All findings were based on statistical analysis of a large infant cohort. Due to limited sample availability, there was no attempt to replicate.
Randomization	M.tb-uninfected controls were matched based on sex, ethnic group, Centre for Disease Control (CDC) weight-for-age percentile and time on study. Considering the big number of samples to be run, they were processed in 6 batches, we ensured that samples from each case and their corresponding matched controls were included in the same run. The assays performed included, in priority order: flow cytometry surface staining for characterisation of T-cells, Enzyme-Linked ImmunoSpot (ELISpot) assay, flow cytometry surface staining for characterisation of B-cells, Mycobacteria Growth Inhibition Assay (MGIA), gene expression and flow cytometry surface staining for characterisation of mucosal-associated invariant T (MAIT) cells. The laboratory researchers performing the assays were blinded to the group allocation.
Blinding	Investigators were blinded to the group allocation during data collection and analysis.

Reporting for specific materials, systems and methods

We require information from authors about some types of materials, experimental systems and methods used in many studies. Here, indicate whether each material, system or method listed is relevant to your study. If you are not sure if a list item applies to your research, read the appropriate section before selecting a response.

Materials & experimental systems

n/a	Involved in the study	
☐	☒	Antibodies
☒	☐	Eukaryotic cell lines
☒	☐	Palaeontology and archaeology
☒	☐	Animals and other organisms
☒	☐	Clinical data
☒	☐	Dual use research of concern

Methods

n/a	Involved in the study	
☒	☐	ChIP-seq
☐	☒	Flow cytometry
☒	☐	MRI-based neuroimaging

Antibodies

Antibodies used

Marker Fluorochrome Clone Volume/ well (ul) Source Cat. No.
1. T cell
Viability Aqua (1/100) N/A 25 Thermofisher L34957
CD3 AF700 UCHT1 1 Thermofisher 56-0038-42
CD4 APC RPA-T4 2 Biolegend 300514
CD8 BV605 RPA-T8 1 Thermofisher 83-0088-42
CD14 PECY7 HCD14 2 Biolegend 325618
CD16 AF488 3G8 2 Biolegend 302019
CD25 APC-CY7 BC96 2 Biolegend 302614
PD1 BV421 EH12.2H7 3 Biolegend 329920
CD127 BV650 A019D5 1 Biolegend 351326
CD56 BV785 5.1H11 1 Biolegend 362550
HLA-DR PE L243 5 Biolegend 307606
CD19 PECY5 B4 1 Biolegend 302210
2. B cell
Viability Aqua (1/100) N/A 25 Thermofisher L34957
CD3 BV510 UCHT1 1 Biolegend 300448
CD19 PECy7 SJ25C1 1 BD Bioscience 557835
CD21 VioBlue HB5 1 Miltenyi 130-101-715
CD27 BV650 O323 2 Biolegend 302828
CD38 PECy5 HIT2 2 Thermofisher 15-0389
CD20 APC-Cy7 2H7 4 Thermofisher 47-0209-42
CD24 FITC SN3 10 Biorad MCA1379F
CD5 APC UCHT2 1 Biolegend 300612
IgD PE IA6-2 5 Biolegend 348204
CD10 BV785 HI10a 3 BD Bioscience 564960
IgG BV605 G18-145 3 BD Bioscience 563246

3. MAIT cell
Viability Aqua (1/100) N/A 25 Thermofisher L34957
CD19 BV510 HIB19 2.5 BD Bioscience 561121
CD3 AF700 UCHT1 1 Thermofisher 56-0038-42
CD4 FITC RPA-T4 2 Biolegend 300506
CD8 APCH7 SK1 2 BD Bioscience 641400
CD14 PECY7 HCD14 2 Biolegend 325618
CD26 PE BA5b 2.5 Biolegend 302706
CD27 BV605 O323 2 Biolegend 302830
Valpha7.2 BV421 3C10 2 Biolegend 351715
CD161 APC HP-3G10 2 Biolegend 339912
CD163 PERCPCY5.5 GHI/61 2 Biolegend 333608
HLADR BV650 L243 2.5 Biolegend 307650

Validation

Source Cat. No. Validation
Thermofisher L34957 https://www.thermofisher.com/order/catalog/product/L34957?SID=srch-srp-L34957
Thermofisher 56-0038-42 https://www.thermofisher.com/antibody/product/CD3-Antibody-clone-UCHT1-Monoclonal/56-0038-42
Biolegend 300514 https://www.biolegend.com/en-gb/products/apc-anti-human-cd4-antibody-823
Thermofisher 83-0088-42 https://www.thermofisher.com/antibody/product/CD8a-Antibody-clone-RPA-T8-Monoclonal/83-0088-42
Biolegend 325618 https://www.biolegend.com/en-gb/products/pe-cyanine7-anti-human-cd14-antibody-3958
Biolegend 302019 https://www.biolegend.com/en-gb/products/alexa-fluor-488-anti-human-cd16-antibody-2734
Biolegend 302614 https://www.biolegend.com/en-gb/products/apc-cyanine7-anti-human-cd25-antibody-1908
Biolegend 329920 https://www.biolegend.com/en-gb/products/brilliant-violet-421-anti-human-cd279-pd-1-antibody-7191
Biolegend 351326 https://www.biolegend.com/en-gb/products/brilliant-violet-650-anti-human-cd127-il-7ralpha-antibody-7673
Biolegend 362550 https://www.biolegend.com/en-gb/products/brilliant-violet-785-anti-human-cd56-ncam-antibody-12129
Biolegend 307606 https://www.biolegend.com/en-gb/products/pe-anti-human-hla-dr-antibody-790
Biolegend 302210 https://www.biolegend.com/en-gb/products/pe-cyanine5-anti-human-cd19-antibody-720
Thermofisher L34957 https://www.thermofisher.com/order/catalog/product/L34957?SID=srch-srp-L34957
Biolegend 300448 https://www.biolegend.com/en-gb/products/brilliant-violet-510-anti-human-cd3-antibody-9792
BD Bioscience 557835 https://www.bdbiosciences.com/en-eu/search-results?searchKey=557835
Miltenyi 130-101-715 https://www.miltenyibiotec.com/GB-en/products/cd21-antibody-anti-human-hb5.html#fitc:30-tests-in-300-ul
Biolegend 302828 https://www.biolegend.com/en-gb/products/brilliant-violet-650-anti-human-cd27-antibody-7660
Thermofisher 15-0389 https://www.thermofisher.com/antibody/product/CD38-Antibody-clone-HIT2-Monoclonal/15-0389-42
Thermofisher 47-0209-42 https://www.thermofisher.com/antibody/product/CD20-Antibody-clone-2H7-Monoclonal/47-0209-42
Biorad MCA1379F https://www.bio-rad-antibodies.com/monoclonal/human-cd24-antibody-sn3-mca1379.html?f=purified&JSESSIONID_STERLING=4718AF2F44410A7F0089F77864F867C5.ecommerce1&evCntryLang=UK-en&cntry=UK&thirdPartyCookieEnabled=true
Biolegend 300612 https://www.biolegend.com/en-gb/products/apc-anti-human-cd5-antibody-868
Biolegend 348204 https://www.biolegend.com/en-gb/products/pe-anti-human-igd-antibody-6532
BD Bioscience 564960 https://www.bdbiosciences.com/en-eu/products/reagents/flow-cytometry-reagents/research-reagents/single-color-antibodies-ruo/bv786-mouse-anti-human-cd10.564960
BD Bioscience 563246 https://www.bdbiosciences.com/en-eu/products/reagents/flow-cytometry-reagents/research-reagents/single-color-antibodies-ruo/bv605-mouse-anti-human-igg.563246
Thermofisher L34957 https://www.thermofisher.com/order/catalog/product/L34957?SID=srch-srp-L34957
BD Bioscience 561121 https://www.bdbiosciences.com/en-eu/products/reagents/flow-cytometry-reagents/research-reagents/single-color-antibodies-ruo/v500-mouse-anti-human-cd19.561121
Thermofisher 56-0038-42 https://www.thermofisher.com/antibody/product/CD3-Antibody-clone-UCHT1-Monoclonal/56-0038-41
Biolegend 300506 https://www.biolegend.com/en-gb/products/fitc-anti-human-cd4-antibody-825
BD Bioscience 641400 https://www.bdbiosciences.com/en-eu/products/reagents/flow-cytometry-reagents/clinical-discovery-research/single-color-antibodies-ruo-gmp/apc-h7-mouse-anti-human-cd8.641400
Biolegend 325618 https://www.biolegend.com/en-gb/products/pe-cyanine7-anti-human-cd14-antibody-3958
Biolegend 302706 https://www.biolegend.com/en-gb/products/pe-anti-human-cd26-antibody-611
Biolegend 302830 https://www.biolegend.com/en-gb/products/brilliant-violet-605-anti-human-cd27-antibody-7804
Biolegend 351715 https://www.biolegend.com/en-gb/products/brilliant-violet-421-anti-human-tcr-valpha7-2-antibody-8878
Biolegend 339912 https://www.biolegend.com/en-gb/products/apc-anti-human-cd161-antibody-7420
Biolegend 333608 https://www.biolegend.com/en-gb/products/percp-cyanine5-5-anti-human-cd163-antibody-4794
Biolegend 307650 https://www.biolegend.com/en-gb/products/brilliant-violet-650-anti-human-hla-dr-antibody-8875

Flow Cytometry

Plots

Confirm that:

- [x] The axis labels state the marker and fluorochrome used (e.g. CD4-FITC).
- [x] The axis scales are clearly visible. Include numbers along axes only for bottom left plot of group (a 'group' is an analysis of identical markers).
- [x] All plots are contour plots with outliers or pseudocolor plots.
- [x] A numerical value for number of cells or percentage (with statistics) is provided.

Methodology

Sample preparation	Cells were stained with antibodies to characterize T, B and MAIT-cells (Supplementary Data 2). To ensure assay reproducibility; antibodies from the same lot were used for staining all samples. Staining was performed in V-bottom 96-well plates, cells were washed in PBS, viability marker diluted in PBS was added cells, which were then incubated for 10 minutes at 4 degree. Surface antibody mix was added and cells were incubated for 30 minutes at 4 degree. Cells were then washed in FACS buffer (PBS, 1% BSA (Sigma) and 0.1% Sodium Azide (Sigma)). Cells were resuspended in 100 ul of 1% Parafolmaldehyde (Aesar) / FACS buffer and were acquired on an LSRII (BD Biosciences), using FACS DIVA (v6.2) and data analysed using Flowjo v8.8 (BD Biosciences). Dead and doublet cells were excluded from the analysis. Fluorescence-Minus one (FMO) gating controls were used. Gating strategies for the 3 panels are illustrated in Supplementary Fig. 1.
Instrument	BD LSRII
Software	Samples were acquired using BD FACSDiva (v6.2) running on Windows (XP 5.1).<br>Post-acquisition analysis was done using Flowjo (BD)
Cell population abundance	No samples sorting was done.
Gating strategy	T-cells (Supplementary Fig. 6a): Singlet, live cells (a-c) were included in analysis. CD3+ cells (d) were used to gate CD4+ and CD8+ T-cells (e). The expression of HLA-DR (1), PD1 (2) and CD25/CD127 (3) was measured on CD4+ (f) and CD8+ (g) T-cells. CD56+, CD56+CD3- and CD56+CD3+ were gated as shown in (h), CD16/CD56 gating is shown in (i). CD14/CD16 populations gating is shown in (j) and expression of HLA-DR on CD14 is shown in (k).<br>MAIT-cells (Supplementary Fig. 6b): Singlet, live CD14-CD3+ T-cells (a-c) were included in analysis. CD3+ cells (d) were used to gate CD4+ and CD8+ T-cells (e). Surface expression of Va7.2, CD26, CD27 and CD161 are shown in plots (f)-(h) and (i)-(k) for CD4+ and CD8+ T-cells respectively. Double positive Va7.2/CD26, Va7.2/CD27 and Va7.2/CD161 are shown in (l)-(n) and (o)-(q) for CD4+ and CD8+ T-cells respectively.<br>B-cells (Supplementary Fig. 6c): Singlet, live CD19+ cells (a-c) were included in analysis. B-cell populations were gated based on the expression of CD27, IgD, IgG, CD5, CD24 and CD38 as shown in plots (d)-(i), details of the subpopulation identification was based on previous studies of B-cell phenotype characterization 1–4.

- [x] Tick this box to confirm that a figure exemplifying the gating strategy is provided in the Supplementary Information.
